# Epstein-Barr virus-encoded LMP2A stimulates migration of nasopharyngeal carcinoma cells via the EGFR/Ca^2+^/calpain/ITGβ4 axis

**DOI:** 10.1242/bio.024646

**Published:** 2017-05-16

**Authors:** Jiezhen Liang, Shixing Zheng, Xue Xiao, Jiazhang Wei, Zhe Zhang, Ingemar Ernberg, Liudmila Matskova, Guangwu Huang, Xiaoying Zhou

**Affiliations:** 1 Department of Otolaryngology-Head & Neck Surgery, First Affiliated Hospital of Guangxi Medical University, Nanning, China, 530021; 2 Department of Otolaryngology-Head and Neck Oncology, The People's Hospital of Guangxi Zhuang Autonomous Region, Nanning, China, 530021; 3 Department of Microbiology, Tumor and Cell Biology (MTC), Karolinska Institutet, Stockholm, Sweden, 17177; 4 Scientific Research Center, Life Science Institute, Guangxi Medical University, Nanning, China, 530021

**Keywords:** Nasopharyngeal carcinoma, Epstein-Barr virus, Latent membrane protein 2, Calpain, Integrin β4, Migration

## Abstract

Epstein-Barr virus (EBV)-encoded latent membrane protein 2A (LMP2A) promotes the motility of nasopharyngeal carcinoma (NPC) cells. Previously, we have shown that the localization of integrin β4 (ITGβ4) is regulated by LMP2A, with ITGβ4 concentrated at the cellular protrusions in LMP2A-expressing NPC cells. In the present study, we aim to further investigate mechanisms involved in this process and its contribution to cell motility. We show that expression of LMP2A was correlated with increased epidermal growth factor receptor (EGFR) activation, elevated levels of intracellular Ca^2+^, calpain activation and accelerated cleavage of ITGβ4. Activation of EGFR and calpain activity was responsible for a redistribution of ITGβ4 from the basal layer of NPC cells to peripheral membrane structures, which correlated with an increased migratory capacity of NPC cells. Furthermore, we demonstrated that the calpain inhibitor calpastatin was downregulated in NPC primary tumors. In conclusion, our results point to LMP2A-mediated targeting of the EGFR/Ca^2+^/calpain/ITGβ4 signaling system as a mechanism underlying the increased motility of NPC cells. We suggest that calpain-facilitated cleavage of ITGβ4 contributes to the malignant phenotype of NPC cells.

## Abstract (precis)

**Summary:** LMP2A expression in nasopharyngeal carcinoma cells increases EGFR activation and cytosolic Ca^2+^, subsequently stimulates calpain-dependent cleavage of ITGβ4 and enhances cell motility.

## INTRODUCTION

Nasopharyngeal carcinoma (NPC) is a prevalent head and neck cancer in Southern China and Southeast Asia (Tao and Chan, 2007). Because of the anatomical location of the nasopharynx and because the early symptoms of NPC are inconspicuous, most NPC patients are diagnosed at an advanced stage, after the appearance of adjacent tissue invasion, lymph node and distant organ metastasis. The undifferentiated NPC is highly sensitive to radiotherapy. With improvement in treatment, the mortality of NPC patients has declined (Chua et al., 2016). However, the development of distant metastasis remains the main cause for treatment failure and death.

Epstein-Barr virus (EBV) infection is closely associated with NPC. Almost 100% of the NPC patients in endemic areas are EBV positive (Pathmanathan et al., 1995). The EBV genome is present and expressed in all NPC cells in patients (Raab-Traub and Flynn, 1986), mainly at type II latency state, expressing two latent membrane proteins (LMP1 and LMP2), EBV nuclear antigen 1 (EBNA1) and EBV-encoded small RNAs (EBERs) (Izumi, 2001). These latent viral messages contribute to the epithelial-mesenchymal transition (EMT) of NPC cells, leading to a more malignant phenotype, which endows the primary tumor with a strong potential to metastasize (Horikawa et al., 2007; Kong et al., 2010; Wang et al., 2014b). It is known that EBV infection activates Ca^2+^-dependent signaling pathways (Chatila et al., 1997; Dellis et al., 2011). By affecting intracellular flux of the ubiquitous second messenger Ca^2+^, EBV plays a fundamental role in progression of EBV-associated tumors. In particular, LMP1 of EBV promotes NPC cell migration and angiogenesis by augmenting store-operated Ca^2+^ entry (SOCE) and transient changes in intracellular Ca^2+^ levels (Dellis et al., 2011; Wei et al., 2015). This enhances the activity of the Ca^2+^-dependent protease, calpain, which catalyzes cleavage of adhesion proteins, such as integrins (Potts et al., 1994; Rintanen et al., 2012), talin (Franco et al., 2004), and focal adhesion kinase (FAK) (Cortesio et al., 2011). Induction of Ca^2+^ fluxes is mediated also by the LMP2A membrane protein (Longnecker, 1998), which is more frequently expressed in NPC tumors than LMP1, but this effect is still incompletely understood. LMP2A is more frequently detected in NPC tumors than LMP1.

In contrast to the ‘eraser’ proteasome system, calpain is regarded as a ‘modulator’ protease. It cleaves proteins at specific sites to modulate their activity, specificity, structures, intracellular localizations, and half-life (Saido et al., 1994; Sorimachi et al., 1997). Calpain expression is altered during tumorigenesis, and the proteolysis of numerous substrates, such as inhibitors of nuclear factor-κB, focal adhesion proteins and proto-oncogenes, has been implicated in tumor pathogenesis (Storr et al., 2011). In line with this, changes in calpain expression were shown to accompany the process of tumorigenesis. Co-existing with calpain is the specific endogenous inhibitor calpastatin (Kawasaki and Kawashima, 1996). Up to now, only the calpain small subunit 1 (CAPN4) was demonstrated as a marker for poor clinical outcome in NPC patients, and was shown to be significantly correlated with EBV infection. CAPN4 promotes NPC metastasis by inducing matrix metalloproteinase 2 expression (Zheng et al., 2014). The transcription of calpastatin and calpain 3 was found to be downregulated in NPC primary tumors, as compared to normal nasopharyngeal epithelium, in cDNA microarray experiments, but no further validation was performed (Sriuranpong et al., 2004). Thus, the functional effects of the EBV latent membrane proteins on the calpain-calpastatin system need to be better understood in NPC.

Previously, we and others have shown that LMP2A stimulates the motility of NPC by its interaction with spleen tyrosine kinase (Syk) (Fotheringham et al., 2012; Zhou et al., 2015a). We also found that the localization of integrin β4 (ITGβ4), a component of hemidesmosomes, was decreased at the basal surface of cells in the LMP2A-expressing NPC cells, but was increased at cellular protrusions (Zhou et al., 2015a). This suggests that LMP2A-induced changes in ITGβ4 localization may influence the migratory dynamics of NPC cells. It was shown that Ca^2+^ signaling is involved in detachment of cells from adhesion structures (Lawson and Maxfield, 1995). Intriguingly, the Ca^2+^-dependent protease calpain was shown to cleave the long cytoplasmic tail of ITGβ4 in *in vitro* cultured cells (Potts et al., 1994), also under conditions not coupled to cell death or starvation. We have previously shown that LMP2A mediates effects on Syk impact on ITGβ4 functions as a structural component of hemidesmosomal adhesive structures and as a transducer of extracellular signaling (Zhou et al., 2015a). It remains to be shown which particular calpain is involved in ITGβ4 cleavage and how this process plays a role in cellular motility. Of relevance is the recent demonstration that the interaction of ITGβ4 and epidermal growth factor receptor (EGFR) is associated with poor prognosis in cancer patients since epidermal growth factor (EGF)-dependent signals stimulate ITGβ4-mediated migration of metastatic cells (Mainiero et al., 1996; Wang et al., 2014a). In addition, it was shown that EGF-induced detachment of trailing edges formed by an ITGβ4 complex in motile cells was partly dependent on calpain activity (Shiraha et al., 1999).

Here, we investigate factors that mediate the effects of LMP2A on the regulation of intracellular calcium levels and how these factors influence ITGβ4 cleavage and movement of NPC cells upon EGFR activation. Our data suggest that calpain is involved in ITGβ4 cleavage, and that this cleavage might be one of the mechanisms responsible for the release of LMP2A-expressing NPC cells from the hemidesmosome-like structures, thus providing a mechanistic correlate to the metastatic behavior of NPC tumor cells.

## RESULTS

### LMP2A-facilitated migration of NPC cells is mediated by an increase in cytosolic Ca^2+^

**Fig. 1.**
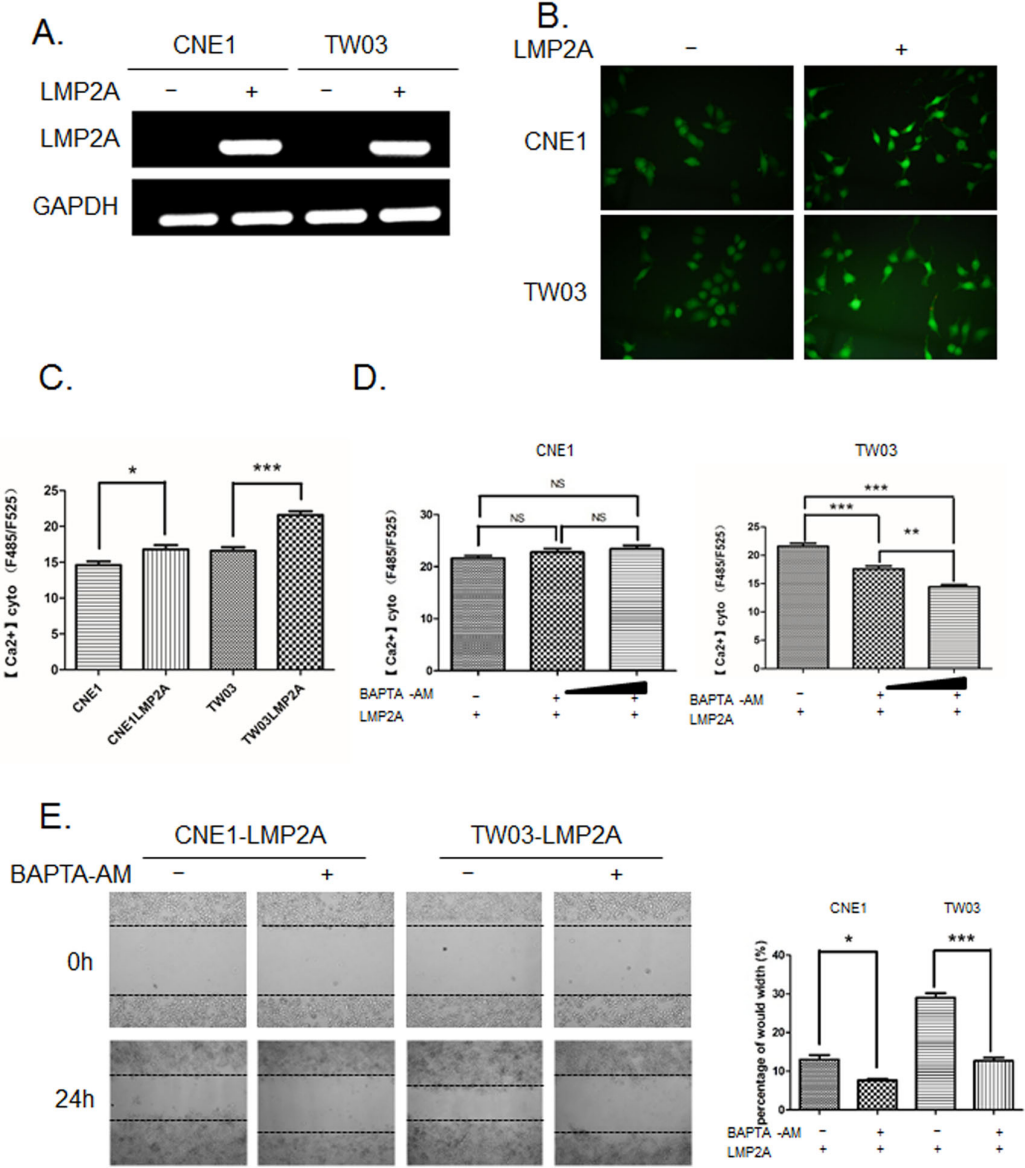
**LMP2A induces an increase in cytoplasmic Ca^2+^ in NPC cells.** (A) MP2A expression in the EBV-negative parental NPC cell lines CNE1 and TW03 (lanes 1 and 3) compared to the corresponding LMP2A-expressing cell lines (lanes 2 and 4) was confirmed by RT-PCR. GAPDH expression was used as an internal control. (B) Fluorescence microscopic images showing the subcellular distribution of fluo3-AM-labeled Ca^2+^ in LMP2A-negative and -positive NPC cell lines (magnification ×40). (C) Fluo3-AM intensity represented the relative amount of Ca^2+^ in LMP2A-negative and -positive CNE1/TW03 cell lines, as recorded by a plate reader. Data are mean±s.d. (*n*=3). (D) Fluo3-AM-labeled Ca^2+^ in LMP2A-expressing CNE1/TW03 cell lines, before and after treatment with a membrane-permeable Ca^2+^ chelator, BAPTA-AM, at 1μm/L and 10μm/L. Data are mean±s.d. (*n*=3). (E) Wound healing assay showing that BAPTA-AM (10μm/L) negatively affects the migratory capacity of LMP2A-expressing NPC cells. The percentage of gap closure was calculated 24 h after scratching. Data are mean±s.d. (*n*=3). *P<0.05; **P<0.01; ***P<0.001; NS, not significant.

To investigate the effect of LMP2A expression on cytosolic Ca^2+^, we established the LMP2A-expressing NPC cell lines LMP2A-CNE1 and LMP2A-TW03 (Fig. 1A). A fluorescent-labeled Ca^2+^ indicator was used to detect the relative amount of intracellular Ca^2+^. In contrast to the parental cell lines, higher Ca^2+^ levels were observed in LMP2A-CNE1 and LMP2A-TW03 (Fig. 1B,C). To address the role of cytosolic Ca^2+^ in cell migration, we used the Ca^2+^ chelator BAPTA-AM to block free Ca^2+^ (Fig. 1D). Upon treatment with BAPTA-AM, both LMP2A-CNE1 and LMP2A-TW03 cells moved slower into the scratch-wounded areas, indicating that cytosolic Ca^2+^ contributes to the motility of LMP2A-expressing NPC cells (Fig. 1E).

### EGFR is activated in LMP2A-expressing NPC cell lines

**Fig. 2.**
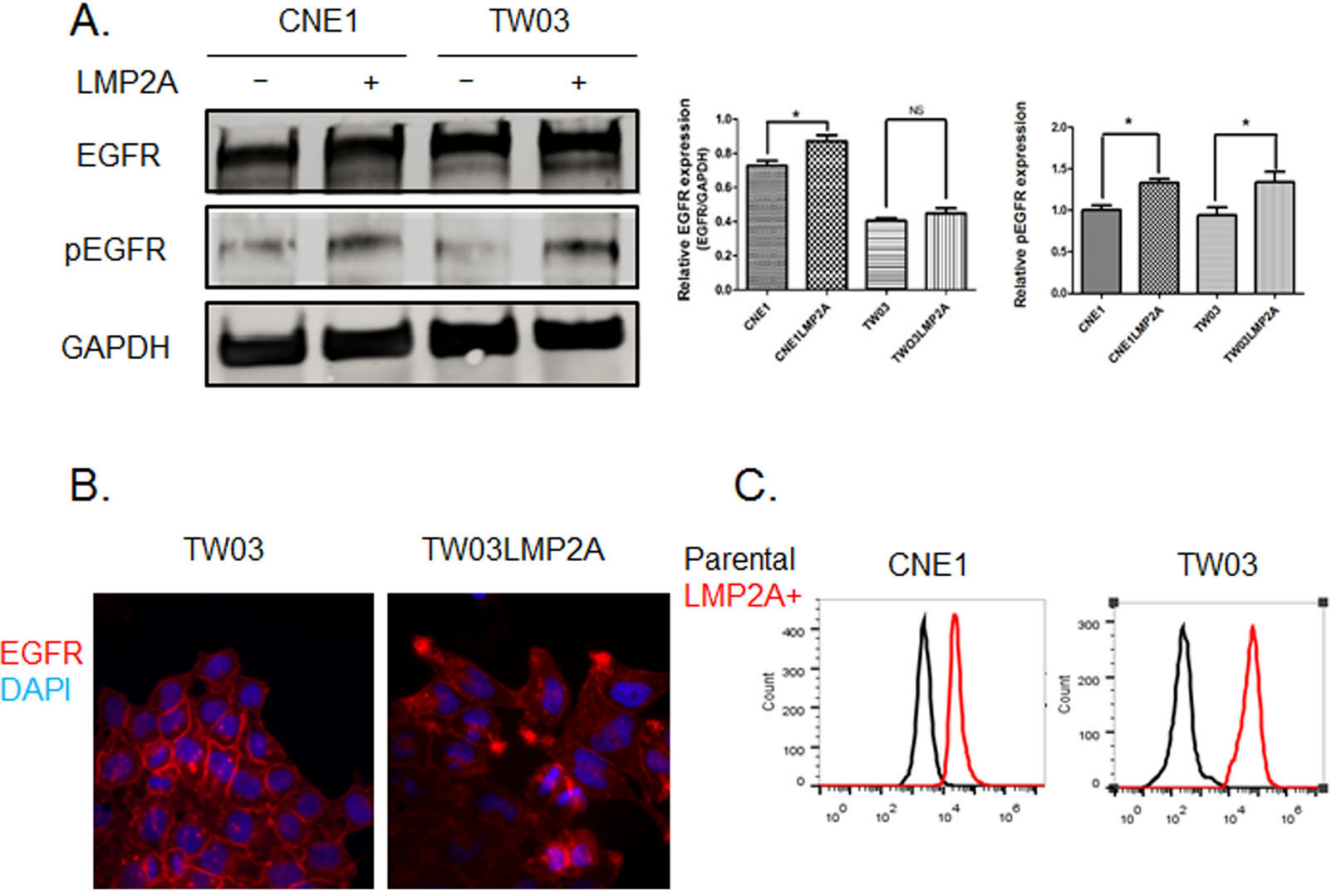
**The expression and localization of EGFR is affected by LMP2A in NPC cell lines.** (A) Western blot analysis of total and phosphorylated EGFR expression in LMP2A-positive and -negative NPC cell lines. Data are mean±s.d. (*n*=3). (B) Immunofluorescence staining of EGFR (red) in LMP2A-expressing TW03 and control cell lines. The nuclei were stained with Hoechst 33258 dye (blue). (C) Flow cytometry analysis of phosphorylated EGFR in LMP2A-expressing CNE1/TW03 (red) and control cell lines (black). Each experiment was performed in triplicate. *P<0.05; NS, not significant.

It has been shown that Ca^2+^ in a complex with calmodulin regulates the activity of EGFR both *in vitro* and *in vivo* (Sánchez-González et al., 2010). EGF stimulation, in turn, increases intracellular Ca^2+^ levels by mediating the extracellular Ca^2+^ entry (Hong et al., 2014). We assessed the correlation between LMP2A expression and EGFR activation, and found that the total expression of EGFR was significantly higher in LMP2A-CNE1 cells than in the parental, LMP2A-negative CNE1 cells. It was also higher in LMP2A-TW03 cells than in TW03 cells, but this difference was not statistically significant (Fig. 2A). However, the membrane localization of EGFR in the LMP2A-TW03 cells was changed. While EGFR was evenly distributed on the cellular membrane of the parental TW03 cells, it was aggregated at the edges of the LMP2A expressing cells (Fig. 2B). This implies an altered functional behavior of EGFR in the LMP2A-TW03 cells. We further analyzed the phosphorylation status of EGFR in the two cell types by western blotting. EGFR was phosphorylated to a greater extent in both LMP2A-expressing NPC cell lines as compared to the parental cell lines (Fig. 2A,C).

### Calpain activity is increased in LMP2A-expressing NPC cell lines

**Fig. 3.**
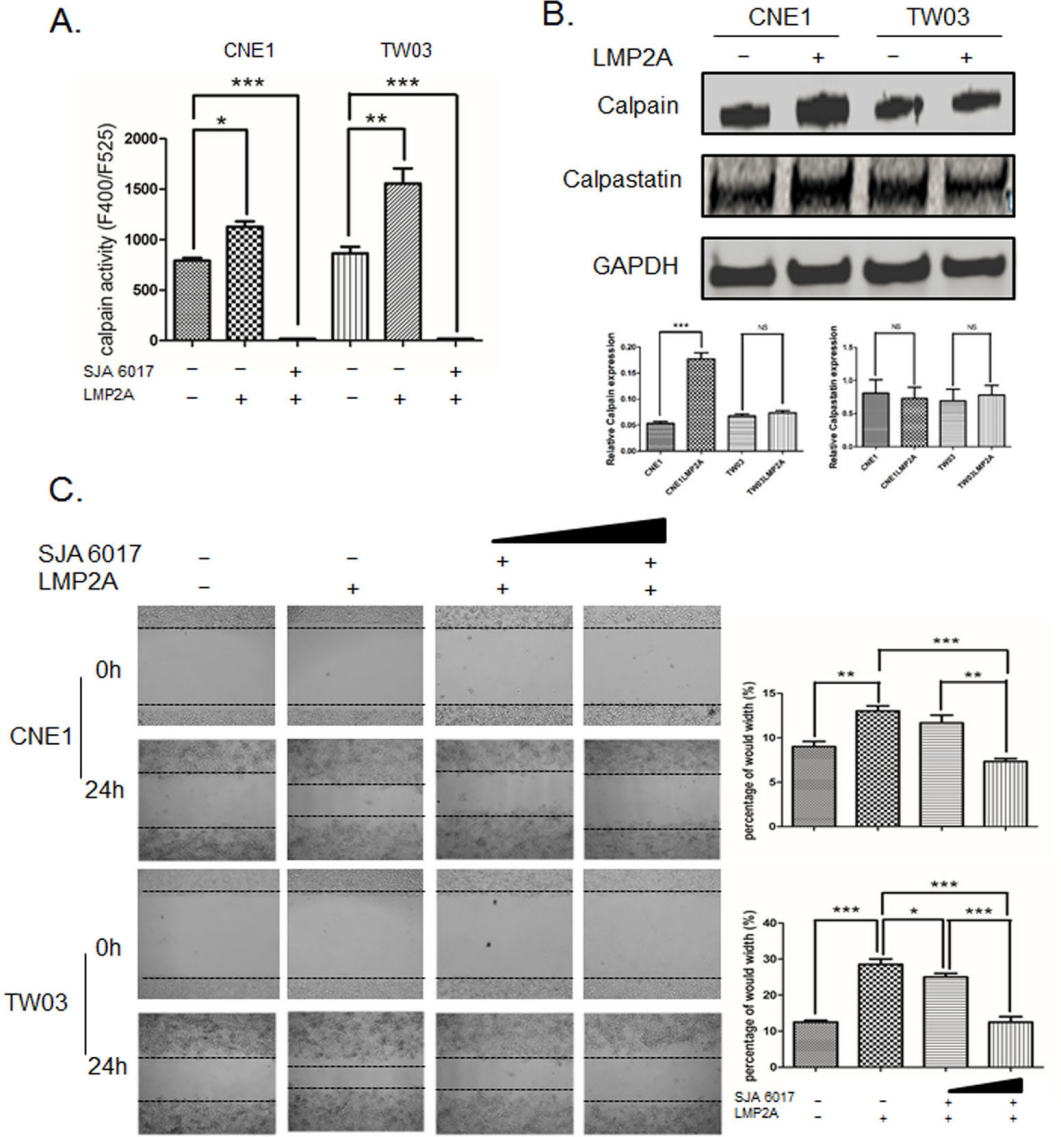
**LMP2A promotes the migration of NPC cells via calpain activity.** (A) Calpain activity was measured in a fluorometric assay in LMP2A-positive and -negative CNE1/TW03 cell lines. A value in cells treated with a calpain inhibitor, SJA6017 (10 ng/μl), for 24 h was used as a baseline. (B) Western blot analysis of calpain and calpastatin expression in CNE1/TW03 cells, with and without LMP2A. GAPDH was used an internal control for protein loading. (C) The motility of LMP2A-expressing CNE1/TW03 cells was inhibited by SJA6017 (5 and 10 ng/μl) in a dose-dependent manner, as assessed by the wound healing assay. The percentage gap closure was calculated 24 h after scratching. Data are mean±s.d. Each experiment was performed in triplicate. *P<0.05; **P<0.01; ***P<0.001; NS, not significant.

Calpain activity was measured in LMP2A-expressing NPC cells and parental control cells. In concordance with our data on cytosolic Ca^2+^ levels, we observed higher calpain activity in LMP2A-CNE1 and LMP2A-TW03 cells as compared to control cells (Fig. 3A). In addition, we analyzed the expression of calpain and the endogenous calpain inactivator calpastatin in LMP2A-positive and control cells. The western blot results showed that the expression of calpain in LMP2A-CNE1 and LMP2A-TW03 cells is increased as compared to the parental cell lines, although the difference was statistically significant for CNE1 cells only (Fig. 3B). We noted, however, that the expression of calpastatin is not affected by LMP2A expression in NPC cell lines (Fig. 3B).

### Calpain-mediated partial cleavage of ITGβ4 contributes to NPC cell migration

To better understand the contribution of calpain activity to the elevated cytosolic Ca^2+^ and increased motility of LMP2A-expressing NPC cells, we employed a calpain inhibitor, SJA6017, in a wound healing assay (Fig. 3C). As shown in Fig. 3C, untreated LMP2A-CNE1 and LMP2A-TW03 cells migrated faster than the parental cell lines. The SJA6017 reagent significantly reduced the motility of these LMP2A-positive NPC cells, and did so in a dose-dependent manner.

**Fig. 4.**
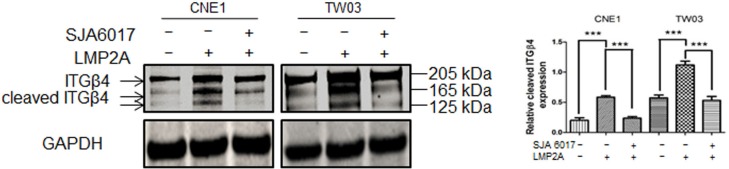
**LMP2A facilitates ITGβ4 cleavage.** Calpain inhibitor reduces the proteolytic cleavage of ITGβ4 in LMP2A-positive cells. Western blot analysis of the full (205 kDa) and cleaved (165/125 kDa) isoforms of ITGβ4 before and after treatment with SJA6017 (10 ng/μl) for 24 h. GAPDH was used an internal control for protein loading. Data are mean±s.d. relative density of cleaved ITGβ4 isoforms (165/125 kDa) (*n*=3). ***P<0.001.

In line with a previous report (Potts et al., 1994), we detected two additional isoforms of ITGβ4, of 165 kDa and 125 kDa molecular weight, in addition to the full-length isoform of ITGβ4 (205 kDa), by western blotting (Fig. 4). Interestingly, the presence of the 165 kDa and 125 kDa isoforms of ITGβ4 was increased in the LMP2A-expressing NPC cell lines, where we also detected higher calpain activity. Treatment of the LMP2A-positive NPC cells with the calpain inhibitor SJA6017 led to disappearance of the low molecular weight isoforms (Fig. 4). This suggests that LMP2A-stimulated NPC cell migration relies on increased cleavage of ITGβ4 by calpain, thus likely interfering with ITGβ4-mediated stabilization of hemidesmosome-like structure.

### Calcium chelator inactivates calpain activity and reduces the cleavage of ITGβ4

**Fig. 5.**
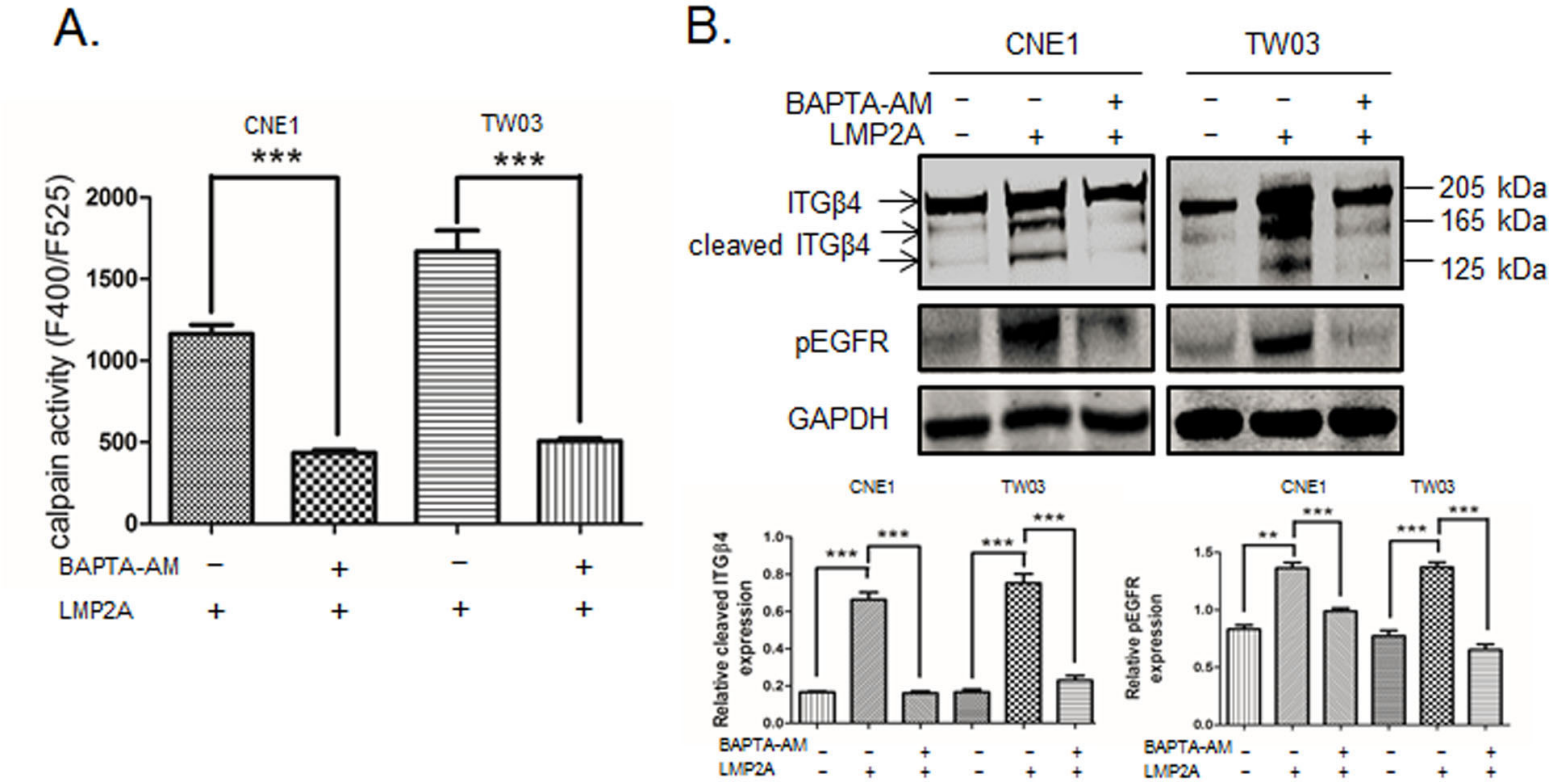
**Calcium chelator inhibits calpain activity and ITGβ4 cleavage in LMP2A-expressing NPC cells.** (A) Calpain activity measured in a fluorometric assay in LMP2A-positive CNE1/TW03 cell lines before and after treatment with BAPTA-AM (10 μm/l) for 24 h. Data are mean±s.d. (*n*=3). (B) Top panel: immunoblots showing the expression of ITGβ4 (205 kDa), cleaved ITGβ4 isoforms (165/125 kDa) and tyrosine-phosphorylated EGFR in LMP2A-expressing and control cells. GAPDH was used as loading control. Bottom panel: semi-quantitative analysis of three repeated immunoblot experiments as in the top panel. Relative expression of the cleaved ITGβ4 isoforms (left) and pEGFR (right) under the same experimental conditions. **P<0.01; ***P<0.001.

We show that the Ca^2+^ chelator BAPTA-AM reduces the motility of LMP2A-expressing NPC cells (Fig. 1D). We also show that the Ca^2+^chelator significantly inhibits the activity of calpain in NPC cells (Fig. 5A). We analyzed the effect of the BAPTA-AM reagent on processing of ITGβ4 by western blotting. Fig. 5B shows that chelating free cytosolic Ca^2+^ not only inhibits the phosphorylation of EGFR, but also reduces ITGβ4 cleavage in LMP2A-CNE1 and LMP2A-TW03 cells.

### EGFR activation increases calpain activity and cleavage of ITGβ4

**Fig. 6.**
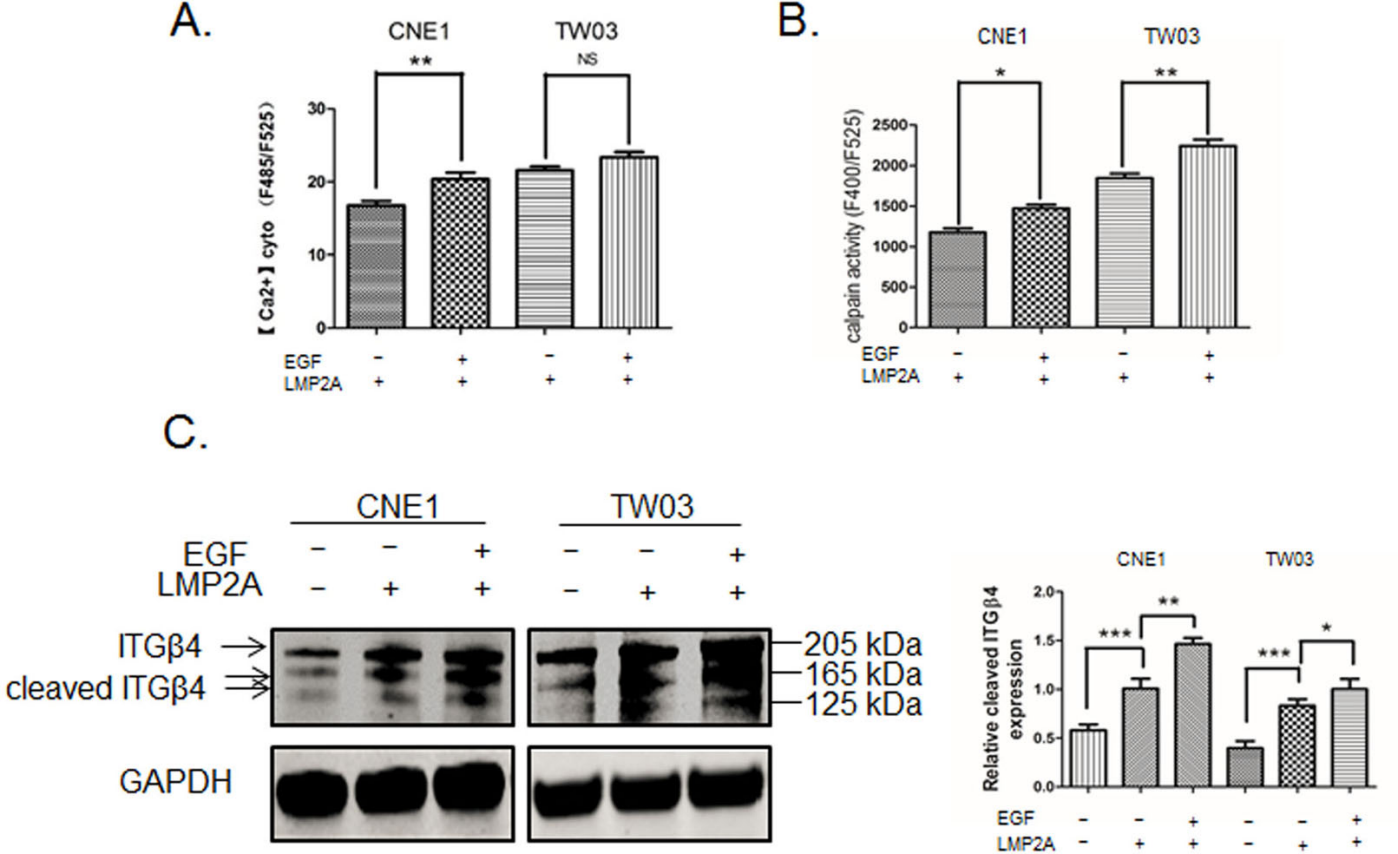
**Activation of EGF receptor increases Ca^2+^, calpain activity and cleavage of ITGβ4.** (A) A Ca^2+^-sensitive fluorescent dye was used to measure cytoplasmic Ca^2+^ in LMP2A-expressing CNE1/TW03 cells after EGF application at 0 and 200 ng/ml. (B) Calpain activity was analyzed in a fluorometric assay in LMP2A-expressing CNE1/TW03 cells after EGF (200 ng/ml) stimulation for 24 h. (C) Western blot analysis of the full (205 kDa) and cleaved (165/125 kDa) isoforms of ITGβ4 after treatment with EGF (200 ng/ml) for 24 h. GAPDH expression was used as an internal control for protein loading. Bar graph demonstrates the relative expression of the cleaved ITGβ4 isoforms. Data are mean±s.d. (*n*=3). *P<0.05; **P<0.01; ***P<0.001.

We stimulated LMP2A-CNE1 and LMP2A-TW03 cells with EGF for 24 h. After stimulation, cytosolic Ca^2+^ content was significantly increased in LMP2A-CNE1 cells (Fig. 6A), and calpain was more activated in both LMP2A-CNE1 and LMP2A-TW03 cells (Fig. 6B). In addition, ITGβ4 protein level increased with EGF treatment and was higher in LMP2A-positive NPC cells than in control cells (Fig. 6C).

### Localization of ITGβ4 at the basal layer of NPC cells is regulated by calpain

**Fig. 7.**
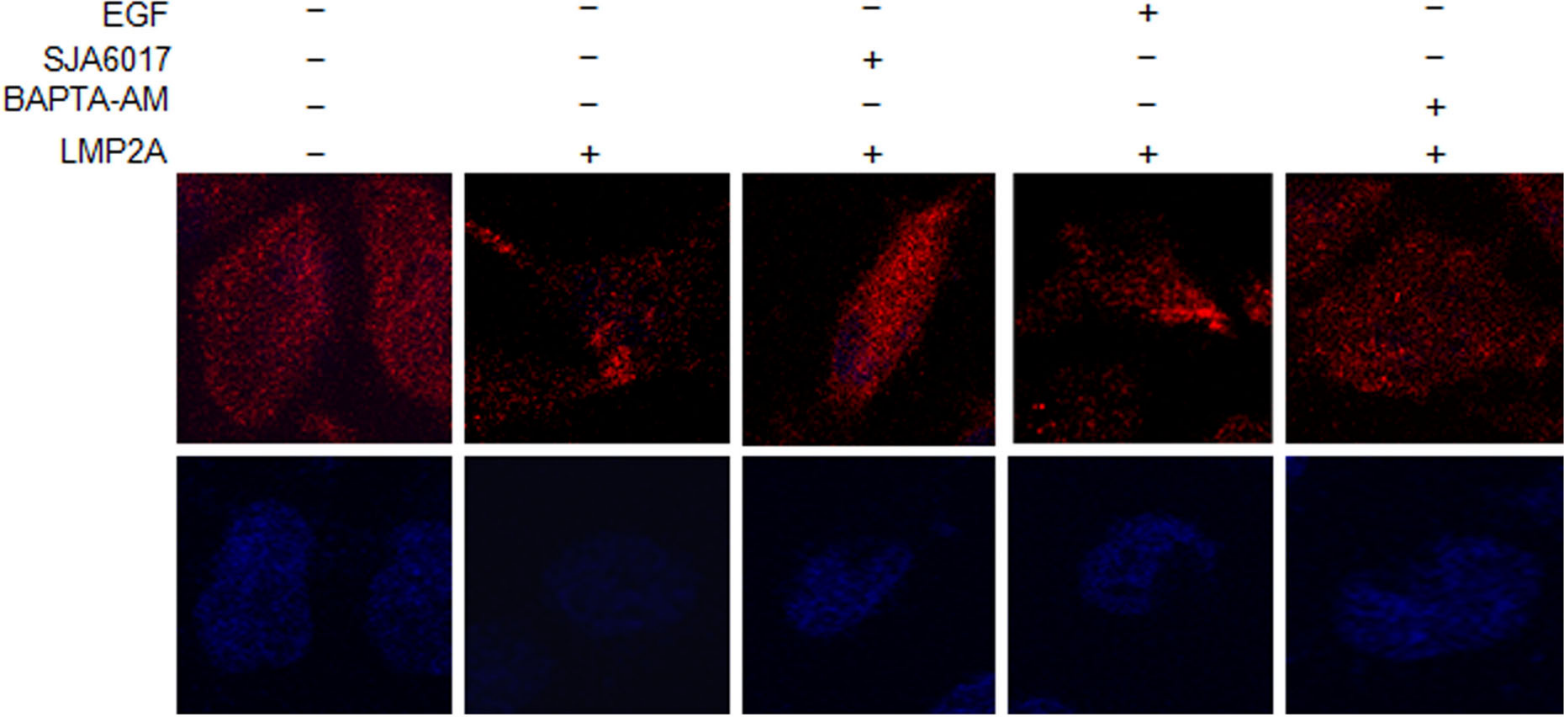
**EGF treatment and inhibition of calpain activity perturbs the distribution of ITGβ4 at the basal layer of NPC cells.** Immunofluorescent staining of ITGβ4 (red) in TW03-parental and TW03-LMP2A cells before and after treatment with EGF (200 ng/ml), SJA6017 (10 ng/μl) and BAPTA-AM (10 μm/l) for 24 h. The nuclei were visualized with Hoechst 33258 (blue) dye.

As we showed previously, ITGβ4 mainly localizes at the basal layer of cells, as a component of hemidesmosome-like structures (Zhou et al., 2015a). In an attempt to clarify the functionality of calpain-mediated ITGβ4 protein cleavage, we analyzed NPC cells stained with ITGβ4 antibody by confocal microscopy. We observed ITGβ4 evenly distributed at the basal cell surface in the slowly migrating parental TW03 cells, while ITGβ4 was aggregated at the cellular edges in the faster migrating LMP2A-positive TW03 cells. We then treated LMP2A-TW03 cells with the BAPTA-AM and SJA6017 reagents, to chelate free Ca^2+^, inactivate calpain and cleave ITGβ4. We observed that the distribution of ITGβ4 at the basal cell surface in the LMP2A-positive cells became similar to the parental TW03 cells. By contrast, stimulation with EGF resulted in ITGβ4 aggregation, which was more pronounced in the LMP2A-positive cells than in parental control cells (Fig. 7). Apparently, this redistribution of ITGβ4 at the cell surface is controlled by calpain activity and reflects a change in motility and metastatic potential of LMP2A-expressing NPC cells.

### The calpain inhibitor calpastatin is inactivated in NPC primary tumors

**Fig. 8.**
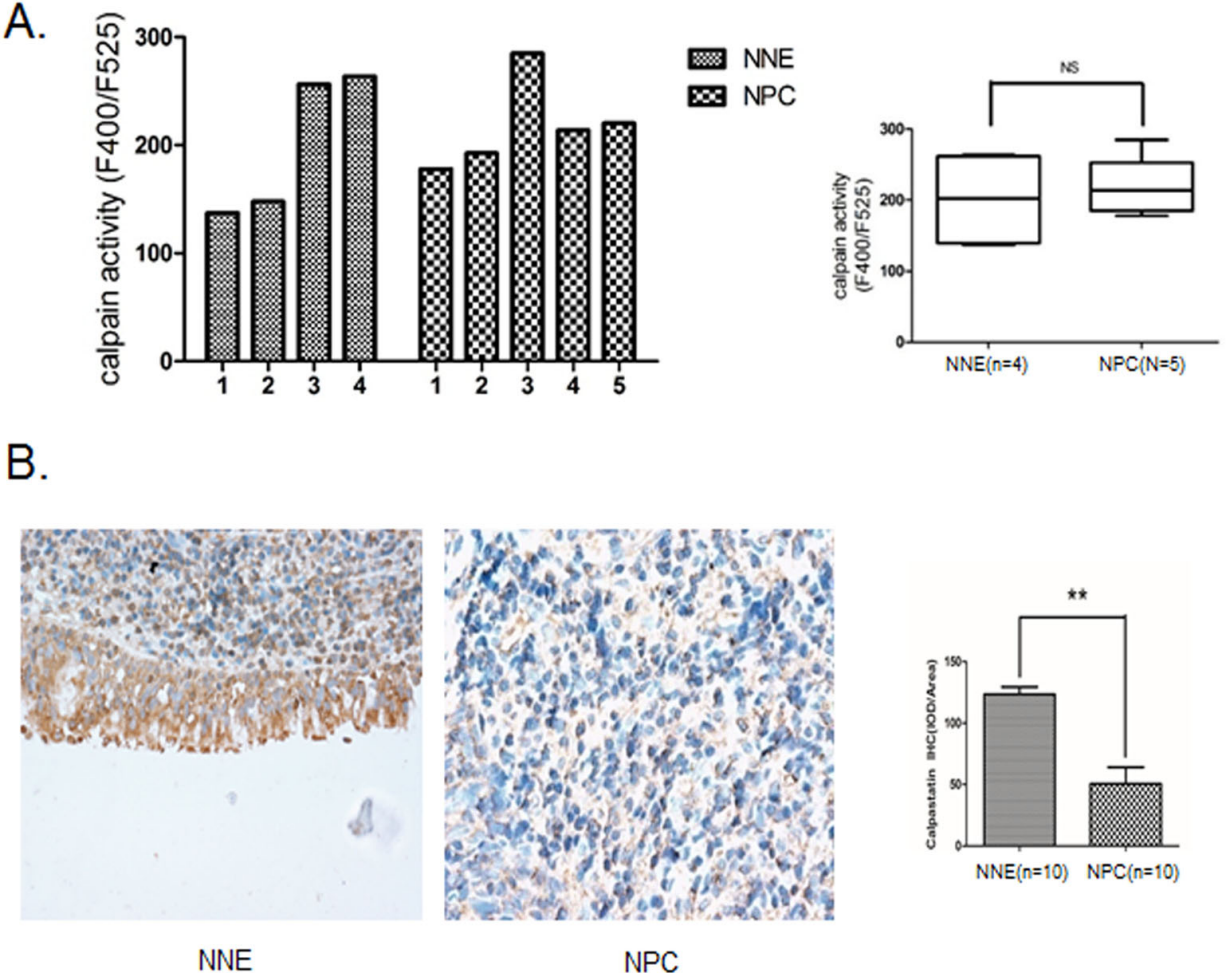
**Calpain activity and calpastatin expression in NPC primary tumor.** (A) Calpain activity was measured in NPC biopsies (*n*=5) and normal control samples (*n*=4) by a fluorometric assay. (B) Immunohistochemical staining of calpastatin in NPC biopsies (*n*=10) and normal control samples (*n*=10; magnification ×400). The integrated optical density (IOD) of each sample was obtained from Image-Pro Plus 6.0 software. Data are expressed as mean±s.d. **P<0.01; NS, not significant.

To evaluate our findings *in vivo*, we analyzed calpain activity in NPC primary tumors and normal nasopharyngeal epithelial cells. As shown in Fig. 8A, mean calpain activity is slightly higher in NPC samples as compared to normal epithelia. The difference is not statistically significant, possibly due to the influence of stroma and adjacent normal cells present in the NPC tissue samples. In addition, we found by immunohistochemical staining that calpastatin was significantly downregulated in the NPC samples (Fig. 8B).

## DISCUSSION

The phenomenon of ITGβ4 cleavage was first described in 1994 (Potts et al., 1994), but its functionality has been questioned from the beginning. Here, we confirm this phenomenon and report that the viral protein LMP2A facilitates ITGβ4 cleavage. We investigate the underlying molecular mechanism and address the functional significance of this process. We show that ITGβ4 cleavage is facilitated by EGFR signaling with an increased intracellular Ca^2+^ level, and is dependent on the activity of calpain. Our data suggest a role for ITGβ4 cleavage as one of the prerequisites for cellular motility. Calpain-mediated cleavage of ITGβ4 regulates the adhesive functions of NPC tumor cells, enabling the dynamic assembly and disassembly of hemidesmosomal adhesive structures.

A common feature of all integrin β subunits is their susceptibility to cleavage by calpain at specific amino acid sequences flanking two closely located NXXY motifs (Pfaff et al., 1999). Calpain-mediated cleavage of the cytoplasmic domain of ITGβ3 controls its bidirectional signaling function (Du et al., 1995). ITGβ4 differs from the other integrin β subunits by a long cytoplasmic domain which contains five NXXY motifs. However, the precise location of calpain cleavage sites in ITGβ4 has not been identified. We demonstrate here that ITGβ4 is cleaved by calpain into 165 kDa and 125 kDa protein subunits. This suggests that the cleavage sites might be located at the NXXY1035 and NXXY1592 positions. We have previously demonstrated that Syk interacts with an ITAM-like motif of ITGβ4 and phosphorylates its tyrosines at positions Y1492 and Y1510. We have also found that LMP2A competes with ITGβ4 for Syk binding. Taken together, this suggests that the reduced tyrosine phosphorylation of ITGβ4 that we observed in LMP2A-positive NPC cells facilitates increased ITGβ4 cleavage (Lu et al., 2006; Zhou et al., 2015a). This is also in line with a report that Syk qualifies as a tumor suppressor because of its ability to reduce calpain activity in breast cancer (Fei et al., 2013). We also have evidence that downregulation of Syk expression was accompanied by increased cleavage of ITGβ4 in the TW03 NPC cell line (data not shown). A negative effect of tyrosine phosphorylation on calpain cleavage of ITGβ3 was previously shown (Xi et al., 2006).

We have observed that it is easier to initiate EGFR signaling in LMP2A-positive NPC cells as opposed to LMP2A-negative control cells. We also find that the localization of EGFR in LMP2A-positive cells is altered in a similar fashion to that of ITGβ4 (Zhou et al., 2015a). We show here that EGF stimulation facilitates proteolytic cleavage of ITGβ4, most likely effecting hemidesmosomal disassembly. In line with previous studies on the intracellular trafficking of ITGβ4, the processed ITGβ4 subunits can be endocytosed and recycled back to the cell surface, with the help of Rab11 and caveolin (Seltmann et al., 2015), to form new adhesive/moving structures. It would therefore be of interest to further investigate the trafficking of ITGβ4 and the effect of LMP2A on this process in NPC cells.

EBV infection reprograms the cellular regulation of Ca^2+^ homeostasis (Dellis et al., 2011; Feng et al., 2012). Here, we demonstrate that LMP2A induces increased intracellular Ca^2+^ levels in NPC cells. Additionally, we have observed that EGF induces higher Ca^2+^ signals in LMP2A-positive cells compared to LMP2A-negative cells (J. W., unpublished data). Cellular movement in itself induces EGFR polarization leading to increased intracellular Ca^2+^ (Wu et al., 2013). Integrin ligands also mediate Ca^2+^ influx from organelles (Umesh et al., 2006). However, the source of Ca^2+^, whether endogenous or exogenous, in stably LMP2A-expressing NPC cells has not been addressed. Thus, blocking the Ca^2+^ flux would be an effective approach for impeding the motility of NPC cells (Zhang et al., 2015, 2013). However, in B lymphocytes, transient expression of LMP2A was shown to block Ca^2+^ mobilization *in vitro* (Chami et al., 2006; Miller et al., 1993). We speculate that, while the transient expression of LMP2A in transfected B cells is extremely high, the stably transformed epithelial cell lines maintain relatively low-level LMP2A expression. A low LMP2A expression level, as we observe in NPC, appears to be associated with increased Ca^2+^ flux and/or Ca^2+^ leakage into cytosol from intracellular stores to elevate the intracellular Ca^2+^. In addition, high LMP2A expression would result in stronger Syk activation, leading to stronger tyrosine phosphorylation of ITGβ4 (Fotheringham et al., 2012) and reduced ITGβ4 cleavage. A low LMP2A expression and low Syk activation would thus enable a fast ITGβ4 turnover and increased cellular motility. Whether ITGβ4 participates in signaling or adhesive cellular complexes (Lakshmanan et al., 2016; Lo et al., 2001) could possibly be studied by altering ITGβ4 tyrosine phosphorylation sites or calpain cleavage sites. Thus, the low level of LMP2A expression in EBV-positive NPC would redirect ITGβ4 localization to promote cell migration and enhance the metastatic properties of the tumor cells.

In conclusion, we found a novel mechanism for LMP2A in promoting motility of NPC cells, via the EGFR/Ca^2+^/Calpain/ITGβ4 axis. The calpain inhibitor calpastatin was inactivated in NPC primary tumors. Calpain-facilitated cleavage of ITGβ4 contributes to the malignant phenotype of NPC. Our data shed light on how the EBV-encoded LMP2A protein might contribute to the metastasis properties of NPC tumor cells and point to possible drug targets for controlling NPC cell migration.

## MATERIALS AND METHODS

### Ethics statement

Ethical permission of this study was approved by the Research Ethics Committee of the First Affiliated Hospital of Guangxi Medical University (Nanning, China).

### NPC cell lines, primary tumors and normal nasopharyngeal epithelia

CNE1 and TW03 cell lines were derived from NPC patients. Cells were cultured in a Dulbecco's modified Eagle medium (DMEM) with 10% fetal bovine serum (Invitrogen) in the presence of streptomycin and penicillin, at 37°C in an atmosphere of 5% CO_2_. LMP2A-expressing CNE1 and TW03 were established as previously described (Zhou et al., 2015a). The expression of LMP2A was confirmed by reverse transcription polymerase chain reaction (RT-PCR).

In total, 15 NPC and 14 normal nasopharyngeal specimens were obtained from the Department of Otolaryngology-Head and Neck Surgery, First Affiliated Hospital of Guangxi Medical University (Nanning, China), after written informed consent from donors, as previously described (Zhou et al., 2015b). They were anonymized for this study. Five NPC and four normal control samples were used for calpain activity measurement. The other biopsies were formalin-fixed and paraffin-embedded for analysis of calpastatin expression.

### Antibodies and reagents

Primary antibodies used were: ITGβ4 (H-101) and calpastatin (H-300) (Santa Cruz Biotechnology); calpain (156, Thermo Fisher Scientific); EGFR (ab52894, Abcam); pEGFR (Tyr1173), calpastatin (4146) and GAPDH (14C10) (Cell Signaling Technology). Secondary antibody 680RD Goat-anti-Mouse and IRDye^®^800CW Goat-anti-Rabbit were from LI-COR Biosciences (Lincoln, NE). Alexa Fluor 488, 594-conjugated antibodies (A11012, A11078 and A11011) were from Thermo Fisher Scientific.

4′-6-diamidino-2-phenylindole (DAPI) was from Vector Laboratories (Burlingame, CA). BAPTA-AM was purchased from Sigma-Aldrich, EGF from Invitrogen, and SJA6017 from Santa Cruz Biotechnology.

### RT-PCR analysis

For gene expression analysis, 2 μg total RNA was used to produce cDNA with use of a Prime Script RT Reagent Kit (Takara) according to the manufacturer's protocols. The resulting cDNAs were amplified with specific primers for LMP2A and GAPDH (Zhou et al., 2015a).

### Measurement of cytosolic calcium content

Cells were seeded on glass slides in 35-mm cell culture dishes overnight and incubated with 5 mΜ Ca^2+^ indicator, fluo3-acetoxymethylester (fluo3-AM), for 30 min in the dark at 37°C. Cells were briefly washed twice in PBS and left for 30 min in PBS before visualization of fluorescently labeled Ca^2+^ under a fluorescent microscope (Olympus, Tokyo, Japan).

### Wound healing assay

Cells were seeded in six well plates at 5.0×10^5^ cells per well and allowed to adhere overnight. A confluent monolayer of cells was scratched with a sterile 1-ml micropipette tip. Plates were then washed twice with culture medium. Images were obtained at the same position of wounding at 0 and 24 h. The wound closures were measured by a light microscope (Olympus, Tokyo, Japan).

### Western blot analysis

Whole-cell lysates were extracted and quantified by using a BCA protein assay kit (Beyotime, Haimen, China). Equal amounts of protein (50 μg) were separated by electrophoresis on 4-12% sodium dodecyl sulfate polyacrylamide gel electrophoresis (SDS–PAGE) gel (Invitrogen) and transferred to nitrocellulose membranes (Thermo Fisher Scientific), which were blocked in 5% bovine serum albumin (BSA) in 1× Tris-buffered saline containing 0.1% Tween-20. Membranes were incubated with primary antibodies at 4°C overnight, then secondary antibodies at room temperature for 1 h. LI-COR Odyssey (LI-COR Biosciences) was used to detect fluorescent signals.

### Flow cytometry

Cells were collected and stained after fixation with 4% formaldehyde for 10 min. After permeabilization using 0.5% Triton X-100, cells were incubated with primary antibodies for EGFR or pEGFR at 1:100 dilution in PBS for 1 h on ice. Secondary Alexa Fluor 488-conjugated antibodies were used at 1:100 dilution for the next 30 min at room temperature. Subsequently, data acquisition was performed using a BD FACSCanto flow cytometer (BD Biosciences) and analyzed by BD FACSDiva software (BD Biosciences).

### Immunofluorescence/confocal microscopy

Cells were grown on cover glasses for 18 h, washed with PBS at 37°C, fixed in 4% formaldehyde for 10 min, permeabilized with 0.5% Triton X-100, and blocked in 5% BSA. Cells were stained with the indicated primary antibodies and appropriate secondary antibodies, and nuclei were counterstained with DAPI. Immunofluorescence images were acquired using a Leica DMRE microscope with HiPic software (Leica, Bensheim, Germany) and a Nikon A1 confocal microscope (Nikon, Tokyo, Japan).

### Calpain activity fluorometric assay

The activity of calpain was determined using a commercial Calpain Activity Fluorometric Assay Kit (BioVision, San Francisco, CA) according to the manufacturer's instructions. Briefly, cells were resuspended and counted, and then 1×10^6^ cells were lysed in 100 μl extraction buffer and incubated on ice for 20 min. An equal amount of cell lysate, 100 μg total protein, was diluted in extraction buffer up to 85 μl, then 10 μl 10× reaction buffer and 5 μl calpain substrate were added, and incubated at 37°C for 1 h in the dark. Luminesence was recorded in a luminometer (GloxMax™ 20/20, Promega, Madison, WI) equipped with a 400 nm excitation filter and a 505 nm emission filter.

### Immunohistochemical analysis

Tissue sections were deparaffinized and rehydrated, to retrieve antigens, then blocked with 5% BSA for 1 h, and incubated with rabbit anti-human calpastatin antibody (1:100 dilution) at 4°C overnight, followed by goat anti-rabbit secondary antibody for 2 h at room temperature. The sections were counterstained with 3,3-diaminobenzidine (DAB-0031/1031, MaxVision, Fuzhou, China) and hematoxylin. Images were acquired under a microscope (Olympus C-5050, Olympus, Tokyo, Japan) and analyzed with Image-Pro Plus 6.0 software (Media Cybernetics, Rockville, MD).

### Statistical analysis

All statistical analyses were performed using GraphPad Prism software, version 5.0 (http://www.graphpad.com/scientific-software/prism/). *P*-values were calculated by unpaired or one-sample *t*-test or one-way or two-way analysis of variance (ANOVA). Specific conditions and *P*-values for each test are described in the figure legends.
